# The Evolution of Board-Certified Emergency Physicians and Staffing of Emergency Departments in Israel

**DOI:** 10.5811/westjem.18541

**Published:** 2024-06-14

**Authors:** Noaa Shopen, Raphael Tshuva, Michael J. Drescher, Miguel Glatstein, Neta Cohen, Rony Coral, Itay Ressler, Pinchas (Pinny) Halpern

**Affiliations:** * Tel Aviv Medical Center, Department of Emergency Medicine, Tel Aviv, Israel; † Tel Aviv Medical Center, Department of Pediatric Emergency Medicine, Tel Aviv, Israel; ‡ Sackler Faculty of Medicine, Tel Aviv University, Tel Aviv, Israel; § Rabin Medical Center, Petach Tikva, Israel, affiliated with the Sackler Faculty of Medicine, Tel Aviv University, Tel Aviv, Israel; ∥ Psychiatry Unit, Sheba Medical Center, Ramat Gan, Israel, affiliated with the Sackler Faculty of Medicine, Tel Aviv University, Tel Aviv, Israel; ¶ Tel Aviv Medical Center, Oncology Division, Psycho-Oncological Service, Tel Aviv, Israel

## Abstract

**Introduction:**

Emergency medicine (EM) was recognized as a specialty in Israel in 1999. Fifty-nine of the 234 (25%) attending physicians working in emergency departments (ED) nationwide in 2002 were board-certified emergency physicians (EP). A 2012 study revealed that 123/270 (45%) of ED attendings were EPs, and that there were 71 EM residents. The EPs primarily worked midweek morning shifts, leaving the EDs mostly staffed by other specialties. Our objective in this study was to re-evaluate the EP workforce in Israeli EDs and their employment status and satisfaction 10 years after the last study, which was conducted in 2012.

**Methods:**

We performed a three-part, prospective cross-sectional study: 1) a survey, sent to all EDs in Israel, to assess the numbers, level of training, and specialties of physicians working in EDs; 2) an anonymous questionnaire, sent to EPs in Israel, to assess their demographics, training, employment, and work satisfaction; and 3) interviews of a convenience sample of EPs analyzed by a thematic approach.

**Results:**

There were 266 board-certified EPs, 141 (53%) of whom were employed in EDs full-time or part-time. Sixty-two non-EPs also worked in EDs. The EPs were present in the EDs primarily during weekday morning shifts. There were 273 EM residents nationwide. A total of 101 questionnaires were completed and revealed that EPs working part-time in the ED worked fewer hours, received higher salaries, and had more years of experience compared to EPs working full time or not working in the ED. Satisfaction correlated only with working part time. Meaningful work, diversity, and rewarding relationships with patients and colleagues were major positive reasons for working in the ED. Feeling undervalued, carrying a heavy caseload, and having complicated relationships with other hospital departments were reasons against working in the ED.

**Conclusion:**

Our study findings showed an increase in the number of trained and in-training EPs, and a decrease in the percentage of board-certified EPs who persevere in the EDs. Emergency medicine in Israel is at a crossroads: more physicians are choosing EM than a decade ago, but retention of board-certified EPs is a major concern, as it is worldwide. We recommend taking measures to maintain trained and experienced EPs working in the ED by allowing part-time ED positions, introducing dedicated academic time, and diversifying EP roles, functioning, and work routine.

Population Health Research Capsule What do we already know about this issue?

*In 2002, 25% of attending physicians working in Israeli EDs were emergency physicians (EP). By 2012, 45% of ED attendings were EPs.*

What was the research question?

*What is the status in 2022? And what factors affect the retention of EPs?*

What was the major finding of the study?

*In 2022, 69% of ED attendings were EPs, but only 59% of all EPs worked in EDs. Part-time employment is a factor in predicting EPs’ satisfaction (OR 9.8, P = 0.02).*

How does this improve population health?

*A nationwide organizational effort is required to maintain trained and experienced personnel working in Israeli EDs.*

## INTRODUCTION

The Israeli Ministry of Health first recognized emergency medicine (EM) as a subspecialty in 1999. Candidates had to be board certified in either anesthesiology, internal medicine, general surgery, family medicine, or orthopedics. Initially, recognition as specialists in EM was issued to 36 selected physicians with long experience and leadership positions working in emergency departments (ED), and the first EM boards exams were offered in 2002.^1^ A national survey conducted in 2002 revealed that only 59 of 234 attending physicians working in EDs nationwide were board certified in EM, and that they were primarily working weekday morning shifts, leaving the ED staffed at other times largely by residents from other specialties. In addition, there were 37 residents in the EM subspecialty residency program.^2^

Emergency medicine was accredited by the Israeli Medical Association Scientific Council as a primary specialty in 2012, and the first residents enrolled in ED training programs. A second national survey conducted during that year showing that 123 of 270 attending physicians employed in EDs nationwide were board-certified emergency physicians (EP). The distribution of the working hours for EPs had remained mostly unchanged compared to the previous decade, with trained EPs primarily working weekday morning shifts. The number of EM subspecialty and specialty residents had risen to 71 in 2012.^3^

In the same year, a labor agreement between the Israel Medical Association, a professional organization representing 95% of Israeli physicians, and the Ministry of Health stipulated the employment mechanism that recognizes the uniqueness of the nature of the work of emergency physicians: a full-time position for EPs was defined as 36 hours per week that may be divided flexibly on weekday mornings and evenings. Additional working hours, as well as night and weekend work, are considered overtime.

Changes in the ED workforce have been seen in recent years in many western countries and have had a major impact on EDs. In the United States, an insufficient number of EPs in the early 2000s seems to have been resolved by the 2020s, at least in urban areas.^4^^–^^6^ In the United Kingdom (UK), an EM staffing crisis induced the establishment of a taskforce, which was able to greatly improve the situation.^7^^–^^10^ We believe that our study can shed some light about the EM staffing crisis, not only in Israel but globally.

Our goals in this study were to re-evaluate the characteristics of the EP workforce in Israel, as well as the employment status and work satisfaction of board-certified EPs working both in and out of the ED. We also surveyed the composition of specialist physicians working in the various EDs in Israel to document the number of board-certified EPs and their workplaces and to examine the factors that influence them to persist in their work in EDs or to move to other areas of practice.

## METHODS

### Study Design and Setting

This was a prospective, cross-sectional study with three components. We conducted a questionnaire-based survey designed to assess the number and percentage of full-time equivalent (FTE), level of training, and specialty (if any) of physicians working in EDs. We enquired about staff member variations at various times during the day as well as during the week (Appendix 1). The survey was adapted from and designed to largely replicate previously published workforce studies of the same population in 2003 and 2012.^2^^,^^3^ The survey was sent to the administrative staff at all 25 Israeli EDs.

An anonymous questionnaire was sent to all 334 board-certified EPs in Israel to retrieve data on their demographics (age, gender, marital status, and number of children); training (years of practice, hospital, and type of residency); place of employment; and work satisfaction. The questionnaire was emailed to all licensed EPs in Israel with the help of the Israel Medical Association. All respondents were asked if they would be willing to participate in an in-depth interview. Those who agreed—EPs employed in EDs and other various fields of practice—created a convenience sample for the third component of the research: a qualitative analysis of in-depth interviews. The interviews were semi-structured, designed by the research team, conducted telephonically, recorded, and transcribed for analysis. Interviewees were asked about their feelings and opinions regarding work in the ED, the field of EM, and their motives for career choices. The qualitative analysis of the data obtained during the interviews was based on a thematic approach. Two independent researchers, both with master’s degrees in psychology, analyzed the data and followed the six phases suggested by Braun and Clarke’s guide for thematic analysis.^11^

### Statistical Analysis

We performed data entry and analysis with SPSS Statistics, version 28 (SPSS Inc, Chicago, IL). Questionnaire response rate was calculated based on the American Association of Public Opinion Research guidelines. We described categorical variables by numbers and percentages, and continuous variables by mean ± standard deviation, median, and interquartile range. Normal distribution was assessed using the Shapiro-Wilk test. We assessed differences in continuous variables between two groups with ANOVA for variances with normal distribution and the Kruskal-Wallis test for variances with non-normal distribution. Differences between categorical variables were assessed with the chi-squared or Fisher exact test, as appropriate, and we assessed differences between medians by a Mann-Whitney U test for independent means. Criteria for satisfaction, which were considered important based on a literature review,^7^^,^^8^^,^^12^ were entered into a multivariate model in which odds ratios and 95% confidence intervals (CI) were calculated for factors found to be significant according to a two-tailed *P*-value of <0.05.

## RESULTS

### National Data and Data from Hospitals

We obtained information from 25/25 Israeli EDs with a survey response rate of 100%, although data on minor points from three EDs was incomplete. There were 266 board-certified EPs of whom 141 (53%) were employed full time or part time in EDs nationwide. Sixty-two non-EP attendings were also employed in the EDs. The average numbers of attendings (both EPs and non-EPs) per ED, stratified by large hospitals (>700 beds), medium hospitals (400–700 beds), and small hospitals (<400 beds), are shown in Table 1.

**Table 1.** Average number of emergency department (ED) attendings employed in Israeli EDs by hospital size in 2021.

Hospital size^*^	Emergency physicians			Non-EP attendings		
	Average number of physicians	Years of practice, mean	FTE fraction, mean	Average number of physicians	Years of practice	FTE fraction mean
Large (n = 10)	8.7	9	0.9	3.6	13	0.8
Medium (n = 7)	3.8	15	1.0	2.3	20	1.0
Small (n = 8)	3.9	8	0.8	6.0	6	0.5

*ED*, emergency department; *EP*, emergency physician; *FTE,* full-time equivalent.

* Large = >700 beds; medium = 400–700 beds; and small = <400 beds.

A FTE is a unit that indicates the workload of an employed person in a way that makes workloads comparable. A FTE of 1.0 is equivalent to a full-time worker, while an FTE of 0.5 represents hours worked that are equivalent to half of those worked by a full-time worker.

**Figure 1.**
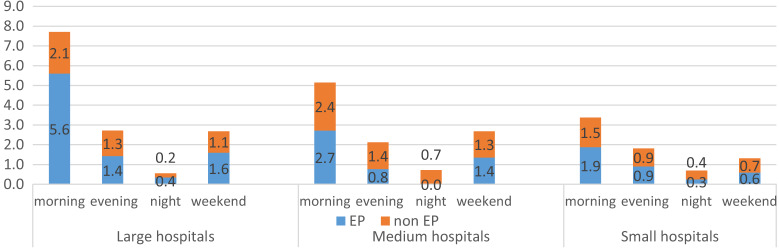
Mean number of emergency department attending physicians by shift. *EP*, emergency physicians.

The presence of EPs in the ED by shift is shown in Figure 1. The EPs were present in the EDs primarily during weekday morning shifts, and their presence was limited during night and weekend shifts, mainly in large hospitals. The numbers of all active EPs, active EPs working the EDs, non-EP attendings working the ED, and EM residents in Israel are shown in Figure 1 and Table 2.

**Table 2.** Israeli physician workforce in emergency departments nationwide, by year.

Year	Active board-certified EPs	Active board-certified EPs employed in EDs^1^	EM residents	Non-EP attendings employed in EDs	Total number of attendings employed in the EDs	Total number of physicians employed in the EDs
2003	59	59	–	175	234	234
2012	154	110	71	147	257	328
2022	239	141	273	62	203	476

*ED*, emergency department; *EP*, emergency physician; *EM*, emergency medicine.

1 Full-time and part-time employment.

### Survey Results

#### Quantitative Analysis

Of 334 questionnaires sent, 106 physicians responded; five responses were excluded due to incomplete replies, for a response rate of 30%. Seventy-nine of the respondents were employed in adults EDs and 22 in pediatric EDs. The mean age of the cohort was 45 ± 10 years, and 65% were males (Table 2). Thirty-five of the 79 EPs in adults EDS (44%) worked full time, 29 (37%) worked part time, and 15 (19%) did not work in any ED. A comparison of age, gender, number of children, residency type, time in practice, and ED weekly hours revealed that they were significantly different between these three groups (*P* < 0.05) (Table 3). Medical experience (in years) was significantly lower among full-time workers compared to both part-timer workers and those who no longer worked in an ED. The mean number of weekly working hours (either in the ED or in another department) was significantly higher for full-timers compared to those who had left the ED. Both total salary and salary per hour were significantly different between the three groups in favor of the group who had left the ED (Table 3).

**Table 3.** Demographics of survey participants.

Variable	Full-time (n = 51)	Part-time (n = 32)	None (n = 17)	*P-*value
Age, years	43.3 ± 7.0	55.0 ± 11.3	56.0 ± 14.4	<0.001
Gender, female, n (%)	18 (38.3)	3 (10.0)	4 (28.6)	0.02
Family status				
Married	7 (13.7)	1 (3.1)	2 (11.1)	
Single	43 (84.3)	27 (84.4)	14 (77.8)	0.19
Divorced	1 (2.0)	4 (12.5)	2 (11.1)	
Children				
<18 years of age	2.1 ± 1.4	1.1 ± 1.4	1.0 ± 1.4	0.004
≥18 years of age	0.57 ± 1.1	2.3 ± 2.0	1.6 ± 1.4	<0.001
Residency path, n (%)				
Direct	8 (15.7)	11 (34.4)	1 (5.6)	
Fellowship	37 (72.5)	11 (34.4)	9 (50.0)	<0.001
By license only	1 (2.0)	10 (31.3)	7 (38.9)	
Years in practice	14.0 ± 7.4	25.6 ± 11.8	26.7 ± 14.9	<0.001
Weekly working hours	39.7 ± 8.8	35.4 ± 15.4	30.0 ± 15.8	0.02
Monthly salary (NIS)^*^	45,400 ± 17,549	73,571 ± 31,530	38,235 ± 17,133	<0.001
Hourly salary (NIS)	248 ± 84	576 ± 570	506 ± 618	0.002

* Average monthly salary in Israel in 2021 – 12,000 NIS.

Values are given mean ±SD unless indicated otherwise.

*NIS*, New Israeli shekel.

### Work Satisfaction

Sixty-nine of the 83 EP respondents who worked in an ED (83%), completed the work- satisfaction section of the survey. The mean age was 49 years (SD 10), 47 (65%) were male, 59 (82%) were married or in a relationship, and 37 (51%) worked full time. Forty physicians reported not being satisfied, and 29 reported that they were satisfied. Table 4 displays a multivariate regression model for ED attending physician satisfaction. Part-time work was the only significant independent predictor of satisfaction, with an adjusted odds ratio of 9.8 (95% CI 1.2–74.9), *P* = 0.02.

**Table 4.** Factors predicting emergency physician job satisfaction.

Variable	aOR (95% CI)	*P*-value
Age (years)	0.9 (0.8–1.0)	0.17
Gender	2.7 (0.4–15.7)	0.26
Family status	1.5 (0.02–97.6)	0.97
Number of children	1.2 (0.5–2.8)	0.63
Part-time position (vs full time)	9.8 (1.2–74.9)	0.02
Emergency physician in adult ED (vs pediatric)	7.8 (0.5–107.4)	0.12
Salary (grade)	1.4 (0.8–2.6)	0.17
ED annual visits	1.0 (0.9–1.0)	0.33

*aOR*, adjusted odds ratio; *ED*, emergency department.

### Qualitative Analysis

Sixty-six of the 83 EP respondents who worked in an ED (80%) completed the survey section on the ED work environment. Most of them reported that they had a heavy workload and a stressful work environment (93% for each). Only 37% felt properly appreciated, and only 41% felt adequately financially compensated. Most of them (81%) had social satisfaction (ie, enjoyed relationship with colleagues), and 75% had professional satisfaction (for further details on work environment in the ED, see Appendix 2).

Thirty-one of the survey respondents who were EPs currently working in an ED (45%) reported considering leaving the ED for various reasons. We compared their reasons with those stated by physicians who had left the ED and, interestingly, few respondents in each group stated that salary was very influential in considering leaving (11%) or in their decision to leave (7%) the ED, despite the major difference in salaries. Lack of opportunity for professional advancement was more influential in the group that was considering leaving (38%) compared to the group that had left (13%). Good social relationships with co-workers was an important factor for staying in the ED, both for those who had left and for those who considered leaving, 70% and 66% (respectively) stating it as “influential” and “very influential.” Work satisfaction was also a significant factor in both groups for staying in the ED (80% and 77%, respectively, stating it as “influential” and “very influential”).

We interviewed 19 EPs who ranged in age from 30–75 years; 12 were male. Sixteen worked in adult EDs and three were pediatric EPs; seven worked full time and three worked part time, and nine had left the ED (two for military service, two for a fellowship program abroad, and one who retired). Of those working in EDs, eight worked in large hospitals (five different hospitals), one in a medium-size hospital, and one in a small hospital. Nine worked in a central hospital, and one worked in a peripheral hospital.

The thematic analysis yielded two major axes: axis 1 in favor of working as an EP, and axis 2 against working as an EP. Each axis had three corresponding themes, and each theme had several sub-themes. (See Table 5 for details on the themes). Three main themes were found on both axes: internal motivational factors; external factors; and relationships. Those findings were in line with results from the quantitative analyses. For example, limited career advancement opportunities were found to be significant in both the quantitative and qualitative analyses (axis 2, theme 2). The two safeguarding factors that emerged in both types of analysis were meaningful work (axis 1, theme 1) and a good relationship with the multidisciplinary ED personnel (axis 1, theme 3).

**Table 5.** Themes of the in-depth interviews with Israeli emergency physicians.

Axis 1	Axis 2
Pros for working as an emergency physician	Cons for working as an emergency physician
1. Internal motivational factors - Meaningful work - Positive previous experience - Personal responsibility - Receiving immediate feedback - Sense of authority - Intellectual satisfaction (learning and teaching opportunities)	1. Internal motivational factors: - Feeling undervalued - Feeling incompatible with role - Effects on one's mental health - Effects on family relationships
2. External factors - Patients and care diversity. - Case-managing - Holistic approach to patient care - Dynamic nature of the field - Suitable compensation for extra hours	2. External factors: - Limited career advancement opportunities - Intense caseload - Verbal and physical abuse from patients and their relatives - Unsuitable baseline wages - Work conditions (staffing, lack of appropriate equipment, lack of sustenance and rest)
3. Relationships - Rewarding patient-doctor relationship - Good relationships with multidisciplinary ED personnel - Good relationships with ED management	3. Relationships - Poor relationships with hospital management - Complicated relationships with consulting experts from other departments - Complicated relationships and tension with other hospital departments

*EP*, emergency physician; *ED*, emergency department.

## DISCUSSION

### The Importance of an Emergency Physician Presence in the ED

It is widely acknowledged that the presence of EPs in the ED is highly beneficial for patient care.^13^ Research carried out in 2014 in a large, urban Israeli medical center found an advantage to the presence of EPs in the ED compared to physicians board-certified in other specialties in terms of length of stay in the ED.^14^ Shortening the patient’s length of stay in an ED reduced ED crowding, a parameter that was found to be associated with reduced mortality.^15^ A study in a rural Australian medical center ED showed improvement in patient wait time and access block (the situation where patients who have been assessed in the ED and require admission are boarded in the ED due to a lack of inpatient bed capacity) when EPs were present.^16^ Another Australian study showed that patients cared for by EM residents benefitted from the presence of an EP attending.^17^ Several UK studies also found clinical benefit in the presence of an EP attending in the ED.^18^^–^^20^ One study noted that senior doctor input in patient care in the ED added accuracy to disposition decisions, thus impacting patient safety and improving departmental flow.^18^ Another study carried out in a pediatric ED showed that the presence of EPs was also cost effective, resulting in fewer admissions, shorter wait time, and fewer patient complaints.^19^ These and other studies promoted a recommendation for 24/7 EP presence in EDs in the UK.^20^

### Emergency Department Clinical Workforce

**Figure 2.**
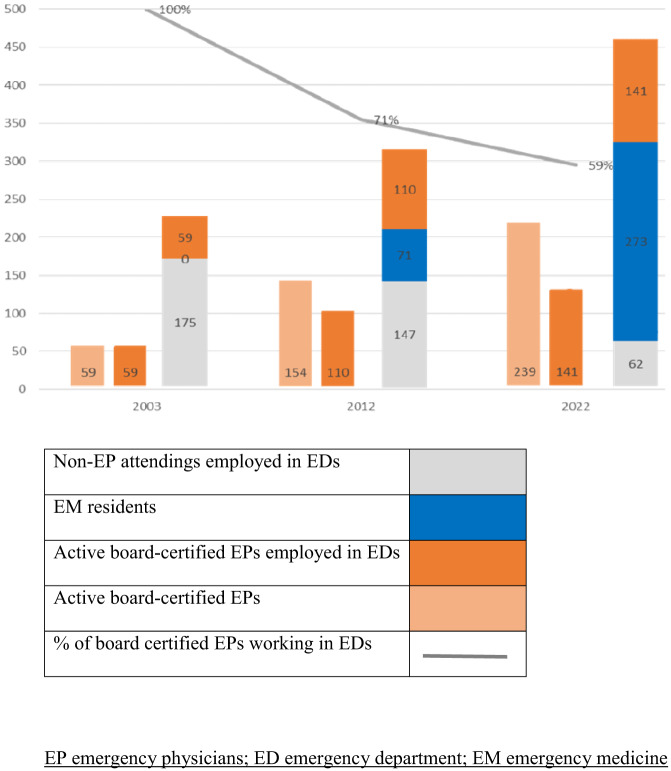
Comparison between workforces in 2003, 2012, and 2022.

We found a decrease in the number of non-EP attending physicians working in EDs nationwide, and a parallel increase in the number of EM residents. There was an increase in the number of EP attendings working in the EDs, but the percentage of board-certified EPs employed in EDs was decreasing, even after considering the number of retired physicians (Figure 2). The two earlier studies on the Israeli ED workforce in 2003^2^ and 2012^3^ found that the presence of EPs in the EDs was mostly limited to weekday morning shifts. The 2012 study showed some presence of EPs on weekends, but only in large (>700 beds) hospitals. The findings of the most recent study, conducted in 2022, showed a similar trend, with an increase in the presence of EPs during morning shifts and a smaller increase, if any, in their presence during evening, night, and weekend shifts. (See Appendix 1 for further details.)

Insufficient numbers of EPs working in EDs were evident in the United States (US) in the early 2000s.^4^ As part of the effort to rectify this shortage, Camargo et al developed a formula calculating the number of EPs required for the proper function of an ED. The calculation was based upon several assumptions: 1) a board-certified EP was present at all times; 2) an average physician can attend 2.8 patients per hour; and 3) there was a 40-hour work week, with one-third of those hours dedicated to non-clinical work. The formula those authors created is:

$$\mathrm{Number}\;\mathrm{of}\;\mathrm{needed}\;\mathrm{doctors}=\frac{\mathrm{annual}\;\mathrm{number}\;\mathrm{of}\;\mathrm{ED}\;\mathrm{visits}}{3548}.$$

Based upon this model, the authors concluded that only 55% of the current EP demand was being met in the US in 2005.^4^ Using data on physicians’ workforce and patient volumes, another group found that the 2016 shortage in EPs in the US was decreasing yearly.^6^ A follow-up study, conducted in 2020, anticipated that the shortage would be resolved as early as 2021, especially in urban zones. Furthermore, that study predicted that, after extrapolating current trends in residency graduation and accounting for increased patient volumes, the EP workforce could be oversupplied by 20–30% by the year 2030.^5^

The specialty of EM suffered a similar staffing crisis in the UK, which led to the establishment of a taskforce dedicated to finding a solution.^7^^,^^8^ The British College of Emergency Medicine established a “rule of thumb” for ED staffing that considered sustainability and the need for resident supervision. According to the proposed guideline, 12–16 certified EPs are required for basic coverage for an ED with 100,000 visits per year, assuming the presence of competent residents and physician assistants.^21^ Following the recommendations of the taskforce, changes in practice and policy, through innovations as well as recognition of the particular stresses posed by a career in EM, led to rapid growth of EM in the UK in terms of both attending physicians and residents.^9^^,^^10^ A shortage of EPs in Australia in 2008 caused some policymakers to advocate for the employment of general practitioners in EDs,^22^ as had been done earlier in Israel (but to a lesser degree after the establishment of an EM residency). Our current study showed similar findings. We, too, observed a major lack in highly trained personnel in the ED, which should eventually be resolved thanks to the increasing numbers of residents in EM.

Several studies found that burnout played an important role in EP turnover.^10^ High burnout rates were also demonstrated in Israeli EPs in a recent study, and that the rate worsened as a result of the COVID-19 pandemic.^23^ Low job satisfaction was linked with leaving and intention to leave the ED, according to other reports.^24^^,^^25^ This issue is a matter of considerable concern: in our current study, 60% of the EPs were found to have low levels of work satisfaction.

The qualitative analysis of our study revealed that the factors contributing to work satisfaction seem to be universal: teamwork; continued training and engaging in academic activities; and work diversity.^26^^–^^30^ Stress and problematic communication with the administration were also found to be negative factors in EP retention.^26^^,^^27^^,^^30^^,^^31^ Our data showed that most EPs find their work to be very stressful. Notably, despite the difference in salaries between EPs who left the ED and those who remained in full- and part-time work and the dissatisfaction with the baseline salaries, salary was rarely the major reason for leaving or considering leaving the ED. This correlates with the finding of our previous study, in which physicians who left reported lower salaries in the ED but did not state that salary was a major reason for leaving.^28^

To the best of our knowledge, the application of a flexible employment model to increase retention in the ED has rarely been discussed in the literature. Part-time employment was suggested by James et al as a means of motivating veteran physicians to continue working in the ED.^29^ In another small, qualitative study, the suitability of EM for flexible working was listed as being a factor influencing the career choice of being an EP.^32^

The concept of part-time work for physicians in general is over two decades old and has been associated with younger and female doctors seeking a better work-life balance. In the late 1990s a series of articles debated the possible impact of flexible and part-time employment on doctors, including its effect on professionalism and career sustainability. The matter of patients’ continuity of care was also debated.^33^^–^^35^ Part-time employment became more common among primary care physicians and pediatricians, but its effect on doctors’ wellbeing and patient outcome was rarely researched.^36^ Parkerton et al found higher quality performance for primary care physicians working part time, and Panattoni et al found higher patient satisfaction.^37^^,^^38^

According to our findings, part-time employment in the ED is an independent predictor for physician satisfaction. Further study is required to determine whether application of this employment model improves work satisfaction and increases retention of EPs. Potentially, a part- time work model could also allow for a larger and more diverse ED workforce.

## LIMITATIONS

This study has several limitations that bear mention. First, we relied upon self-reported data for the EDs. Secondly, the questionnaire had a relatively low (30%) response rate and was subject to response bias and under-representation of various groups: our findings showed that while 41% of EPs are not employed in EDs, only 19% of the survey respondents belonged to the group of EPs who had left the ED, rendering that group under-represented in the survey. Other, less easily identified groups may also be under-represented. Additionally, the in-depth interviews were conducted with a small convenience sample and were thus subject to selection bias.

## CONCLUSION

Emergency medicine in Israel is at a crossroads. On the one hand, a larger than ever number of young doctors have chosen EM for their residency training. On the other hand, the retention of board-certified EPs is a major concern. It is our view that a nationwide organizational effort is required to maintain trained and experienced clinicians working in our EDs.

## Supplementary Information




