# Supplementary material for: The Evolution of Board-Certified Emergency Physicians and Staffing of Emergency Departments in Israel
Source: West J Emerg Med. 2024 Jun 14;25(4):584–92. doi: 10.5811/westjem.18541 (PMC11254163; doi:10.5811/westjem.18541)
Supplement: Supplementary file 1 [file wjem-25-584-s001.docx]

**Appendix Legends**

1. Appendix 1: Phases of thematic analysis
2. Appendix 2: ED physician workforce nationwide by year
3. Appendix 3: Attending physicians per shift in the years 2002, 2012, and 2022, and in large, medium, and small hospitals
4. Appendix 4: Demographics of survey participants included in the work satisfaction multivariable analysis
5. Appendix 5: Work environment in the ED
6. Appendix 6: Analysis of reasons for leaving the ED
7. Appendix 7: Detailed thematic analysis

ED emergency department

**Appendix 1 Phases of thematic analysis**

The researchers first familiarized themselves with the material by reading and re-reading the transcripts. Preliminary ideas were then written down and initial codes were generated from each individual transcript. The transcripts were then systematically coded and grouped together into elevated categories, based on being similar or because they belonged to the same conceptual content. These categories were sorted into themes and overarching themes. This involved grouping some categories and generating new themes to better define the scope of the categories. The themes were then reviewed by and debated among the researchers to check whether some could be combined, refined, or discarded for lack of cohesion. The most descriptive quotes were selected when the researchers reached full agreement regarding each theme.

| **Phase** | **Description** |
| --- | --- |
| 1. Familiarization | Transcribing the interviews, reading, and re-reading the transcripts allowed for initial ideas to be conceptualized. |
| 1. Generating initial codes | Coding interesting features in a systematic manner across all transcripts, grouping together details relevant to each code. |
| 1. Searching for themes | Sorting the codes into potential themes and overarching  themes. Gathering all data relevant to each theme. |
| 1. Reviewing themes | Reviewing the identified themes. Checking to see whether themes could be combined, refined, or discarded. Generating a thematic outline of the analysis. |
| 1. Defining and naming themes | Continued analysis to refine themes, generating clear definitions and names for each theme. |
| 1. Producing the report | Selection of the most descriptive examples, final analysis of the selection |
| *Note.* Adapted from Braun & Clarke (2006). | |

**Appendix 2. Israeli ED physician workforce nationwide, by year**

| Year | Active board-certified EPs | Active board-certified EPs employed in EDs^1^ | EM residents | Non-EP attendings employed in EDs | Total number of attendings employed in the EDs | Total number of physicians employed in the EDs |
| --- | --- | --- | --- | --- | --- | --- |
| 2003 | 59 | 59 | - | 175 | 234 | 234 |
| 2012 | 154 | 110 | 71 | 147 | 257 | 328 |
| 2022 | 239 | 141 | 273 | 62 | 203 | 476 |
| ^1^Full-time and part-time employment | | | | | |  |

**Appendix 3: Comparison of attending physicians per shift in 2002, 2012 and 2022 in large, medium, and small hospitals.**

The date shows an increase in the number of EPs present during the morning shift in all hospitals, with a concurrent decease in the number of non-EP attendings. The increase in the presence of EPs in other shifts is modest, if any. The EPs presence during night and weekend shifts is scarce in all hospitals, and their presence during evening shifts is mostly limited to large hospitals.


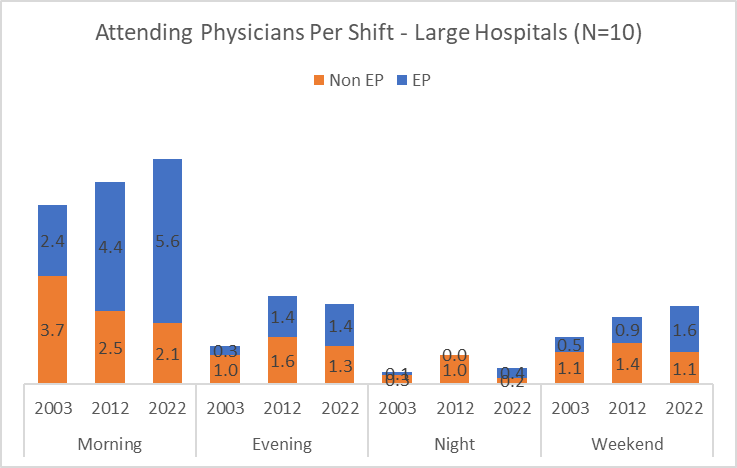


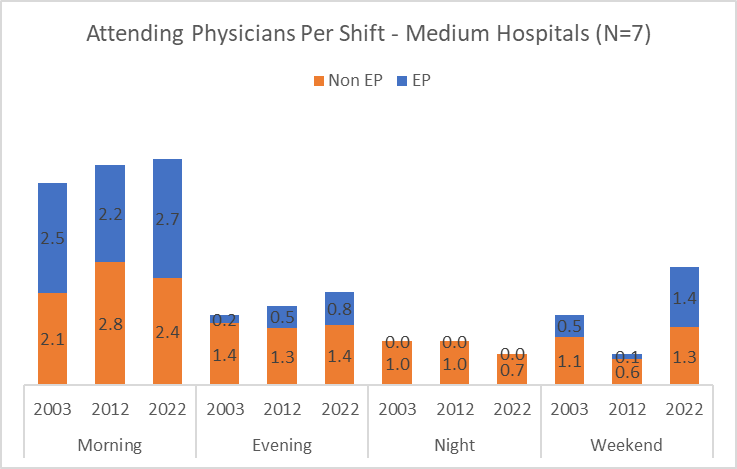


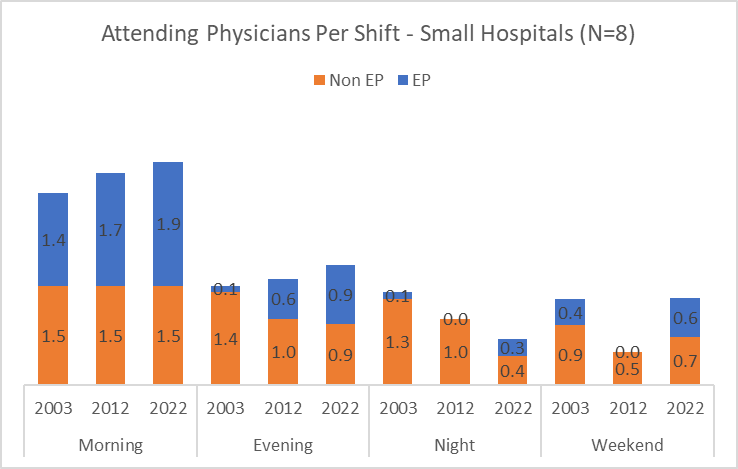


**Appendix 4: Demographics of survey participants included in the work satisfaction multivariable analysis**

| Variable | N= 69 |
| --- | --- |
| Age (mean±SD) | 49±10.8 |
| Sex, males (%) | 47 (65.3) |
| Family status: 1  2  3 | 6 (8.3)  59 (81.9)  4 (5.6) |
| Children <18 years, n (mean±SD) | 1.5±1.3 |
| Full/partial time: Full  Partial | 37 (51.4)  31 (43.1) |
| Geographical location  Center  Periphery | 47 (65.3)  12 (16.7) |
| Salary, in NIS (mean±SD) | 27,000±17,000 |
| ED annual visits, median [IQR] | 94,500 [53,000-108,000] |

SD standard deviation; NIS New Israeli Shekel

**Appendix 5**: **Work environment in the ED**

In total, 66 EPs completed the part of the survey on work environment in the ED. Over 74% of the participants take part in teaching medical students and 89% take part in teaching residents. Forty-one attendings (64%) reported participating in research during the 5 years before the survey.

| N=66  (N, %) | Heavy workload | Stressful | Properly appreciated | Adequate financial compensation | Professional development avenues | Social satisfaction | Professional satisfaction |
| --- | --- | --- | --- | --- | --- | --- | --- |
| Very true | 44 (67%) | 42 (64%) | 12 (18%) | 10 (15%) | 9 (14%) | 24 (36%) | 24 (37%) |
| True | 17 (26%) | 19 (29%) | 25 (38%) | 17 (26%) | 17 (26%) | 30 (45%) | 25 (38%) |
| Partially true | 2 (3%) | 2 (3%) | 15 (23%) | 25 (38%) | 17 (26%) | 5 (8%) | 10 (15%) |
| Slightly true | 2 (3%) | 2 (3%) | 7 (11%) | 8 (12%) | 12 (20% | 3 (5%) | 3 (5%) |
| Not true | 1 (2%) | 1 (2%) | 7 (11%) | 6 (9%) | 10 (15%) | 4 (6%) | 3 (5%) |

**Appendix 6: Analysis of reasons for leaving the ED**

Seventeen participants were EPs who left the ED altogether. Among the 69 EPs who remained in the ED, 31 (45%) reported considering leaving the ED, and 27 of them specified their reasons for doing so.

| Reasons for leaving /staying | Salary | | Lack of professional advancement possibilities | | Good social connection with co-workers | | Work satisfaction | |
| --- | --- | --- | --- | --- | --- | --- | --- | --- |
| Groups | Left the ED (N=15) | Considering leaving (N=27) | Left the ED (N=15) | Considering leaving (N=27) | Left the ED (N=10) | Considering leaving (N=27) | Left the ED (N=915) | Considering leaving (N=27) |
| Very influential | 1 (7%) | 3 (11%) | 2 (13%) | 10 (38%) | 5 (50%) | 9 (33%) | 10 (67%) | 12 (45%) |
| Influential | 2 (13%) | 6 (22%) | 1 (7%) | 7 (26%) | 2 (20%) | 9 (33%) | 2 (13%) | 9 (33%) |
| Partially influential | 2 (13%) | 4 (15%) | 2 (13%) | 3 (12%) | 2 (20%) | 5 (19%) | 2 (13%) | 1 (4%) |
| Slightly influential | 3 (20%) | 5 (19%) | 2 (13%) | 0 (0%) | 0 (0%) | 1 (4%) | 1 (7%) | 4 (15%) |
| Not influential | 7 (47%) | 9 (33%) | 8 (53%) | 6 (23%) | 1 (10%) | 3 (11%) | 0 (0%) | 1 (4%) |

Despite significant differences in salaries, salary was rarely mentioned as a major factor for leaving the ED by those physician who left (7%) or considered leaving (11%). Lack of professional advancement possibilities was a much more prominent consideration among those who considered leaving than among those who actually left. Work satisfaction and good relationship with coworkers were important reasons for staying in the ED for both groups.

**Appendix 7: Detailed thematic analysis**

| Axis 1: Pro | |
| --- | --- |
| Comments | Themes |
|  | Theme 1: Internal motivational factors |
| "I get to save lives – after years of work I still feel fulfilled following successful resuscitation…or whenever I find a solution for patients…and they leave the ER satisfied" (D1). | Meaningful work |
| "When I started my studies I realized that was my field, and even as a student I used to love working in the ER." (D8). | Positive previous experience |
| "I was fond of the integrative approach, for example, when I was responsible for a variety of ER triage patients" (D16). | Personal responsibility |
| "[I chose to specialize in this field] because I was passionate for and interested in the professional content, and because of the real-time feedback for my interventions" (D12). | Receiving immediate feedback |
| "When you experience the chaos of the ER, and you manage to control it, you get a sense of satisfaction [from it]" (D1). | Sense of Authority |
| "You get to be the first physician to meet the patient and figure out the riddle that the patient presents" (D12). | Intellectual satisfaction |
|  | Theme 2: External factors |
| "First of all, there is great case diversity, in terms of a variety of diseases and injuries, work is never really boring….We get to do a bit of internal medicine, surgery, some orthopedics…" (D2). | Diversity |
| "…I prefer being the case-manager, where you are more or less the physician in charge of both the acute and the chronic stages" (D16). | Case managing |
| "I was interested in a holistic field of medicine where you aren't focused on just one organ" (D15). | Holistic approach to patient care |
| "I favor the fast rhythm of work – you have to make quick [medical] decisions as soon as the door opens" (D18). | Dynamic nature of the field |
| "The compensation for extra hours accumulates to a decent salary" (D6). | Suitable compensation for extra hours |
|  | Theme 3: Relationships |
| "Being there for the patient and their family during their time of crisis, and being able to ease their experience, is a positive aspect of my work as an ER physician" (D12). | Rewarding patient-doctor relationship |
| "I am most gratified when I am in the company of my ER team. I believe that in them the ER finds its greatest asset and that is what makes all the difference" (D19). | Good relationships with multidisciplinary ER personnel |
| "They understand the residents' needs and treat us well" (D14).  "My supervisors were wonderful" (D6).  "I had a good relationship with the ER management" (D17). | Good relationships with ER management |

| Axis 2: Cons | |
| --- | --- |
| Comments | Themes |
|  | Theme 1: Internal motivational factors |
| "It was an intense experience. There were moments where I hit rock bottom, particularly when I was met with patients' ingratitude. I was treated like their punching bag, and I felt like the hospital's gatekeeper" (D10).  "I felt like we were constantly under scrutiny. Our professional opinion was distrusted and we were disrespected" (D17). | Feeling undervalued |
| "As residents, we didn't delve into other relevant specialties, which left us feeling undertrained and unschooled" (D17). | Feeling incompatible with role |
| "I treasure the work in the ER, however, residency was a traumatic experience. It is a period where you are supposed to be learning and shaping your professional future, but working long shifts, both day and night, adds to the overall feeling of stress" (D15). | Effects on mental health |
| "Being a resident, and simultaneously becoming a parent, was overwhelming. Particularly having to work long shifts. The day before your shift you are anxious and the day after you are exhausted. The following day you have to return to work [and the cycle continues]. It wasn't a pleasant period of time" (D15). | Effects on family relationships |
|  | Theme 2: External factors |
| "There is a shortage of career advancement opportunities and sub-specialties in the field of emergency medicine" (D11). | Limited career advancement opportunities |
| "In the last decade we have seen a major increase in caseload in the ER, which we experience around the clock" (D16). | Intense caseload |
| "I've witnessed several violent incidents…Someone threw a computer screen at a young doctor. It happens a lot and it's scary. I've also seen an attack by a younger patient on an older paramedic, which resulted in a concussion to the latter" (D8).  "Patients and their relatives [sometimes] have unrealistic expectations that the ER will solve all their problems, and they can get very aggressive toward the medical staff" (D13). | Verbal and physical abuse from patients and their relatives |
| "The basic salary is disgraceful" (D4). | Unsuitable baseline wages |
| "There is a general lack in equipment, space, staff, and ETC" (D6).  "The work conditions were disagreeable – from the food in the cafeteria, to the absence of suitable rooms for resting" (D8). | Work conditions (staffing, lack of appropriate equipment, lack of palatable food and place to rest) |
|  | Theme 3: Relationships |
| "I quit because I realized, at a certain point, that the behavior of my supervisors (hospital management) was harming my health" (D3). | Poor relationships with hospital management |
| "The ER typically requests consultations from other departments, but it is not a reciprocal relationship. So, as a young doctor, compared to my consulting colleagues, I felt a little less informed and barely tolerated as somewhat of a step-sibling" (D17). | Complicated relationships with consulting experts from other departments |
| "We are not doing other departments any favors – we only increase their workload. So, that's why it is especially difficult to gain a favorable view from the department heads" (D5).  "The funds for positions in other departments were reallocated [by management] to our department. [From that point onwards] it looked like the other departments were rooting for us to fail" (D9). | Complicated relationships and tensions with other hospital departments |
